# The Association between Body Mass Index and Health-Related Quality of Life in Treatment-Seeking Arab Adults with Obesity

**DOI:** 10.3390/medsci6010025

**Published:** 2018-03-13

**Authors:** Leila Itani, Simona Calugi, Riccardo Dalle Grave, Dima Kreidieh, Germine El Kassas, Dana El Masri, Hana Tannir, Aya Harfoush, Marwan El Ghoch

**Affiliations:** 1 Department of Nutrition and Dietetics, Faculty of Health Sciences, Beirut Arab University, P.O. Box 11-5020 Riad El Solh, Beirut 11072809, Lebanon; l.itani@bau.edu.lb (L.I.); d.kraydeyeh@bau.edu.lb (D.K.); g.elkassas@bau.edu.lb (G.E.K.); dana.masri@bau.edu.lb (D.E.M.); h.tannir@bau.edu.lb (H.T.); Aya_harfoush@hotmail.com (A.H.); 2 Department of Eating and Weight Disorders, Villa Garda Hospital, Via Montebaldo, 89, 37016 Garda, Italy; si.calugi@gmail.com (S.C.); rdalleg@gmail.com (R.D.G.)

**Keywords:** BMI, obesity, Arab states, Middle East, health-related quality of life, ORWELL 97

## Abstract

Few studies have thus far been carried out on Health-Related Quality of Life (HRQoL) and obesity in Arab-speaking countries, an issue that we therefore set out to investigate in this study. HRQoL was assessed by the validated Arabic version of the ORWELL 97 questionnaire in 129 treatment-seeking individuals with obesity referred to the Nutritional and Weight Management Outpatient Clinic at the Department of Nutrition and Dietetics of Beirut Arab University (BAU) in Lebanon, and 129 normal-weight participants of similar age and gender. Participants with obesity, regardless of gender, displayed higher total ORWELL 97 scores when compared with normal-weight controls, indicating that obesity is associated with lower HRQoL. Linear regression analysis showed that a higher body mass index (BMI) is associated with an increase in ORWELL 97 scores, but only among female, not male, participants with obesity (β = 2.89, 95% confidence interval (CI) = 1.43–4.53, *p* < 0.001). Moreover, logistic regression analysis showed that a one unit increase in BMI increases the odds of an ORWELL 97 score ≥ 71.75—considered indicative of a clinically significant impairment of HRQoL—by nearly 23% (odds ratio (OR), 95% CI = 1.23, 1.09–1.40, *p* < 0.05). If confirmed, our findings should prompt clinicians operating in Arab countries to encourage patients with obesity to initiate and persevere in weight-loss programs at the earliest opportunity.

## 1. Introduction

Since the early 80s, there has been growing interest in a new dimension of quality of life, termed Health-Related Quality of Life (HRQoL)—defined as an assessment of how the individual’s wellbeing may be affected over time by a disease, disability or disorder [1]. Research focused on HRQoL is considered important, and has been identified as a primary outcome because of its influence on current and future treatments and health protocols [2]. For example, several studies have shown that HRQoL is reduced in obesity [3]. Indeed, obesity is associated with major medical and psychosocial comorbidities [4,5], and people with obesity are expected to experience sub-optimal health for a much higher proportion of their life than those without [6].

Although obesity is a health problem that is increasing worldwide [4], the growing body of evidence regarding HRQoL in obesity that has emerged over the past three decades is mainly derived from Western populations [3]. Patients in Western countries have therefore benefitted considerably from the resulting information on the impact of this condition on the functioning and wellbeing of the individual, which has helped researchers to evaluate and improve the effects of treatments [7]. In Arabic-speaking countries, on the other hand, despite the fact that the prevalence of obesity is dramatically growing—especially in the Gulf Cooperation Council countries (Bahrain, Kuwait, Oman, Qatar, Saudi Arabia and the United Arab Emirates—very little is known about HRQoL in Arab adults with obesity [4,8]. The few available studies that have assessed HRQoL in obesity relied on non-specific weight-related HRQoL questionnaires known for their inability to accurately assess the impact of obesity on quality of life [9]. Indeed, reliable tools have not, until recently, been validated for the Arabic-speaking population [10,11]. 

However, a recent study validated the Arabic version of the ORWELL 97—a specific questionnaire designed to assess obesity-related quality of life—and found it suitable for use in Arab adults with obesity [12]. For this reason, we set out to use ORWELL 97 to investigate the impact of obesity on HRQoL, and assess any association between HRQoL and body mass index (BMI), in a group of Arabic-speaking treatment-seeking adults with obesity. This preliminary study was designed to provide some insight into the association between obesity and HRQoL in this population.

## 2. Materials and Methods

A cross-sectional study was conducted between May 2017 and January 2018 on 258 participants. The sample size was determined to be 230, using a two-sided test at the 0.05 levels and a power of 80% and a minimum change in ORWELL 97 score of 10 units and standard deviation of 27 [13]. A 20% non-response rate was assumed, and the total target sample size was 276 (138 participants: 138 comparison group). The patients were recruited consecutively by quota recruitment until an adequate sample size was attained. One hundred and twenty-nine participants with obesity (43 males and 86 females) seeking weight-loss treatment were recruited. All treatment-seeking participants had been referred by general practitioners to the Nutritional and Weight Management Outpatient Clinic at the Department of Nutrition and Dietetics of Beirut Arab University (BAU) in Lebanon. The inclusion criteria were: (i) the ability to follow instructions on how to fill out the questionnaires; (ii) BMI ≥ 30 kg/m^2^; and (iii) age ≥ 18 years. The only exclusion criterion was that the participant should not be undergoing current weight-loss treatment or have achieved significant weight loss (≥10% in kg) in the last six months.

One hundred and twenty-nine normal-weight participants (41 males and 88 females) with BMI ≥ 18.5 and ≤24.9 kg/m^2^ were recruited from the general population in various community settings as a comparison group, through a simple randomized community e-mail-based survey sent to members of the BAU and other mailing lists. All participants were recruited consecutively, and filled out the questionnaire at one time point (before treatment in the case of the clinical sample). All reported data in the study pertain to one-point data collection.

The study design was reviewed and approved by the Institutional Review Board of Beirut Arab University (No. 2017H-0034-HS-R-0241), and all participants gave informed written consent for the use of their anonymous personal data.

### 2.1. Demographics and Clinical Status

A questionnaire was administered to both test participants and controls to retrieve information regarding their medical history, and demographic and social conditions (age, gender, geographical region, and marital status).

### 2.2. Measures

Body weight was measured to the nearest 0.1 kg in the clinical sample using an electronic weighing scale (SECA 2730-ASTRA). Height was measured to the nearest 0.5 cm using a stadiometer. Height and weight were self-reported in the community sample. The BMI of each participant was then determined according to the standard formula of body weight (kg) divided by height (m) squared.

The validated Arabic version of the ORWELL 97 questionnaire [12] used to assess the participants’ HRQoL is composed of 18 items designed to examine the intensity and subjective relevance of obesity-related physical and psychosocial distress; for each item the participant is asked to score, on a four-point Likert scale, the occurrence and/or severity of the symptom (occurrence) and the subjective relevance of the symptom-related impairment in their life (relevance) [13]. The score of the item is calculated as the product of occurrence and relevance. The global ORWELL 97 score is obtained as the sum of the scores given for individual items—higher global ORWELL 97 scores indicate a lower obesity-related quality of life. A global score on the ORWELL 97 questionnaire of ≥71.75, corresponding to the 75th percentile of the population, was considered indicative of a clinically significant burden on obesity-related quality of life [14]. Accordingly, the global ORWELL 97 score was dichotomized as either “clinically significant” or “not clinically significant” based on the 75th percentile of the population score.

### 2.3. Statistical Analysis

Frequencies, medians and interquartile ranges were used to describe the sociodemographic and anthropometric characteristics of the treatment-seeking individuals with obesity and normal-weight controls. Medians and frequencies were compared using the Mann–Whitney U test and Fisher’s exact test, respectively. Simple and multiple linear regression analyses were used to assess the variation in global ORWELL 97 score with variation in BMI and other covariates. Diagnostic tests of the regression assumption for linearity and equal variance of residuals, and the variance inflation factor (VIF) for testing collinearity between independent variables were conducted. Quantile regression analysis was also conducted to study the effect of the independent variables in different quantiles of ORWELL 97 scores. Simple and multiple binary logistic regression models were used to assess the association between BMI and HRQoL in terms of the increase in the odds of outcome per unit increase in BMI. In this analysis, the outcome measure was “clinically significant” or “not clinically significant”, based on the 75th percentile of the population. Simple logistic regression analyses were run separately for each independent predictor, and a combined model was subsequently performed. The Hosmer–Lemeshow test was used to test logistic regression model fit. In parallel to logistic regression a Poisson regression with robust standards errors was used to estimate the relative risk (RR). All analyses were conducted using the Statistical Package for Social Sciences (IBM Corp. Released 2015. IBM SPSS Statistics for Windows. Version 23, Armonk, NY, USA). Results were considered statistically significant at *p* < 0.05.

## 3. Results

The median and interquartile range of age and gender were similar in treatment-seeking individuals with obesity (33.0 (25.0–46.0) years) and normal-weight controls (34.7 (28.7–41.5) years), with the majority of both participants (68.2%) and controls (66.7%) being female. Among participants with obesity 38.0% met the criteria for class I obesity, 37.2% for class II obesity and 24.8% for class III obesity (data not shown).

Table 1 presents the sociodemographic and anthropometric characteristics and global ORWELL 97 scores of the clinical sample, split by males and females and whether they scored above or below the 75th percentile of the population on the ORWELL 97 (considered indicative of a clinically significant burden on HRQoL or not, respectively). Demographic variables (age and gender) were similar in clinical and control groups (Table 1). Compared to normal-weight participants, both male (9.00 (4.0–16.5) vs. 40.00 (29.0–57.0)) and female (11.00 (6.0–18.0) vs. 42.5 (24.5–73.3)) treatment-seeking participants with obesity had significantly higher median global ORWELL 97 scores (Table 1). Simple linear regression analysis showed that the independent variables age and presence of chronic disease were not correlated with HRQoL in either males or females with obesity. However, BMI was significantly correlated with HRQoL in females with obesity, although it was not correlated with HRQoL in males with obesity. Multiple linear regression analysis of females with obesity showed that, among these participants, a one-unit increase in BMI was associated with an almost three-unit increase in global ORWELL 97 score (β = 2.89, 95% confidence interval (CI) = 1.43–4.53, *p* < 0.001) (Table 2). The multiple quantile regression analysis showed that age did not affect HRQoL in the median and highest quantiles of HRQoL (Table 2). Age alone fitted into quantile regression showed no significant influence on HRQoL in different quantiles (data not shown).

Moreover, combined logistic regression analysis showed that BMI is a predictor of a clinically significant impairment in quality of life (global ORWELL 97 score ≥ 71.75) among females, but not males, with obesity (results for males are not shown (the *p*-value for the interaction term = 0.008). Regardless of the obesity class, in females with obesity, for each one-unit increase in BMI, the odds of scoring ≥71.75 on ORWELL 97—indicative of a clinically significant impairment in obesity-related quality of life—increased by 23% (odds ratio (OR), 95% CI = 1.23, 1.09–1.40, *p* < 0.05) (Table 3). A Poisson regression with robust standard error and adjusting for age and presence of chronic disease, revealed almost no increase in risk (relative risk (RR), 95% CI = 0.99, 0.86–1.13) with increase in BMI in males, and a 13% increased risk (RR, 95% CI = 1.13, 1.08–1.19) in females.

## 4. Discussion

The ORWELL 97 questionnaire is an 18-item measure of obesity-related quality of life that has been validated in Italian-, English- and Portuguese-speaking populations [13,15,16]. The recently validated Arabic version [12] enabled us to carefully assess, apparently for the first time, HRQoL in Arabic-speaking treatment-seeking individuals with obesity, an investigation that produced two major findings.

The first finding revealed that treatment-seeking Arab adults with obesity had a lower health-related quality of life when compared with normal-weight controls. In addition, a significant association was found in females (but not in males) with obesity between higher BMI and a major impairment in obesity-related quality of life, as measured by the ORWELL 97. To date, our study is the first to report such finding in Arabs, and it is therefore difficult to compare our data with previous findings in this population. A few studies have assessed quality of life in Arab adults with obesity, but various methodological limitations such as uncontrolled design and/or quality of life being assessed as a secondary outcome make it difficult to make meaningful comparisons [10,11]. However, our finding is in line with data from a large Korean National Survey that included more than 13,000 adult males and females and showed a significant association between abdominal obesity and reduced HRQoL among females, but not among males [17]. Although examining this gender difference in the association between BMI and HRQoL was beyond the scope of our study, it may be due to the effect of psychological and sociocultural factors on females. In particular, the impact of obesity on psychological wellbeing may not be the same in males and females [18], and further research should be conducted to investigate this apparent gender-related difference. Indeed, the second finding from our study is that, regardless of obesity class, even a small increase, of 1 unit, in BMI increases the odds of a clinically significant impairment in quality of life (≥71.5 units = 75th percentile of the population) by around 23 % in treatment-seeking females with obesity. Once again, this is the first time that such data has been presented in this population.

Our study has some limitations. Primarily, our sample included only individuals seeking outpatient weight-management treatment, and our findings are therefore not generalizable to individuals with obesity seeking other types of treatment (e.g., bariatric surgery or weight-loss drugs). Secondly, the cross-sectional design of the study and the relatively small sample size mean that our results will require replication in a larger sample, and preclude any firm conclusions being drawn about the association between obesity and HRQoL. Nonetheless, since our findings derive from results with acceptable confidence intervals, they can be interpreted in terms of clinical significance.

Future larger-scale longitudinal observational studies are, however, required in treatment-seeking populations with obesity in clinical settings in other Arab countries. The aims of these studies should be to assess obesity-related quality of life using validated instruments such as the ORWELL 97 to identify factors that are strongly associated with HRQoL (i.e., psychological, eating behaviors etc.) in individuals with obesity, and to detect any modification in HRQoL (i.e., improvement/deterioration) in relation to weight changes (i.e., loss, regain) during weight-management programs. It will be also important to compare findings from such studies with those derived from treatment-seeking males and females with obesity in Western countries, to assess whether cultural factors play a role in mediating the effect of obesity on HRQoL.

To this end, our group, in collaboration with other researchers from 12 Arab countries (Algeria, Bahrain, Egypt, Iraq, Kuwait, Lebanon, Morocco, Palestine, Saudi Arabia, Sudan, Tunisia, UAE) and one Italian clinical unit, aims to assess HRQoL in a large sample of treatment-seeking patients with obesity presenting for treatment in specialized weight-management centers in these countries. The aim of this “Quality of Life in individuals with Obesity living in Middle East and North Africa (QoLOMENA)” study is to identify the factors (i.e., psychological and eating behaviors) associated with HRQoL, and to document the modification of the latter in relation to the weight changes achieved during weight-management programs.

## 5. Conclusions

This study indicates that obesity is associated with impaired HRQoL in Arabic-speaking treatment-seeking individuals with obesity, and that higher BMI is associated with a lower HRQoL in this population, but only in females. In fact, even a one-unit increase in BMI may dramatically increase the risk of HRQoL impairment to a clinically significant level. If confirmed, these results should stimulate clinicians operating in Arab countries to encourage patients with obesity to initiate and persevere in weight-loss programs at the earliest opportunity.

## Figures and Tables

**Table 1:** Sociodemographic characteristics, body mass index (BMI) and global ORWELL 97 scores in the study population split by males and females (*n* = 258).

Total Study Sample (*n* = 258)												
	Males (*n* = 84)						Females (*n* = 174)					
	Normal Weight Comparison Group	Patients with Obesity		Participants < 75th Percentile of ORWELL 97 Score ^1^	Participants ≥ 75th Percentile of ORWELL 97 Score ^2^		Normal Weight Comparison Group	Patients with Obesity		Participants < 75th Percentile of ORWELL 97 Score ^1^	Participants ≥ 75th Percentile of ORWELL 97 Score ^2^	
Variables	(*n* = 41)	(*n* = 43)	*p*-Value	(*n* = 35)	(*n* = 8)	*p*-Value	(*n* = 88)	(*n* = 86)	*p*-Value	(*n* = 64)	(*n* = 22)	*p*-Value
Age (years)	32.40 (9.45)	33.00 (16.00)	0.714	33.00 (16.00)	25.50 (19.25)	0.063	35.80 (13.55)	33.50 (22.50)	0.173	32.00 (24.75)	37.50 (20.50)	0.510
Marital status			0.492			0.217			0.003			0.006
Single	16 (39.0)	13 (30.2)		9 (25.7)	4 (50.0)		27 (30.7)	46 (53.5)		40 (62.5)	6 (27.3)	
Married	25 (61.0)	30 (69.8)		26 (74.3)	4 (50.0)		61 (69.3)	40 (46.5)		24 (37.5)	16 (72.7)	
Presence of chronic disease			<0.001			0.432			<0.001			0.803
No	39 (95.1)	24 (55.8)		21 (60.0)	3 (37.5)		75 (85.2)	50 (58.1)		38 (59.4)	12 (54.5)	
Yes	2 (4.9)	19 (44.2)		14 (40.0)	5 (62.5)		13 (14.8)	36 (41.9)		26 (40.6)	10 (45.5)	
Residence			0.273			1.000			0.047			1.000
Urban	31 (75.6)	37 (86.0)		30 (85.7)	7 (87.5)		83 (94.3)	72 (84.6)		53 (84.1)	19 (86.4)	
Rural	10 (24.4)	6 (14.0)		5 (14.3)	1 (12.5)		5 (5.7)	13 (15.3)		10 (15.9)	3 (13.6)	
BMI (kg/m^2^)	23.04 (2.42)	37.48 (8.72)	<0.001	38.19 (7.26)	33.69 (10.49)	0.151	21.98 (2.86)	36.44 (6.12)	<0.001	34.92 (5.96)	38.82 (5.43)	0.002
ORWELL 97 total score	9.00 (12.50)	40.00 (28.00)	<0.001	36.00 (26.00)	84.00 (28.25)	<0.001	11.00 (12.00)	42.50 (48.75)	<0.001	34.00 (30.50)	98.00 (33.75)	<0.001

^1^ <75th percentile of ORWELL 97 score not clinically significant burden; ^2^ ≥75th percentile of ORWELL 97 score clinically significant burden; Values for continuous variables are expressed as median and interquartile range (IQR), and categorical variables as *n* (%).

**Table 2:** Linear regression coefficients and 95% confidence intervals (CIs) for ORWELL 97 and among patients with obesity seeking treatment.

							**Females (*n* = 86)**									
												**Quantile Regression**				
	**Simple linear regression model**						**Multiple linear regression model**					**25th**		**Median**		**75th**
**Independent Variables**	**β**	**Standardized β**	***p*-Value**	**95% CI**	***R*^2^**	**β**	**Standardized β**	***p*-Value**	**95% CI**	***R*^2^**	**β**	***p*-Value**	**β**	***p*-Value**	**β**	***p*-Value**
BMI	2.88	0.394	<0.001	1.42–4.34	0.155	2.89	0.40	<0.001	1.43–4.35	0.179	0.55	0.359	3.40	0.005	4.24	0.001
Age (years)	−0.29	−0.11	0.30	−0.83–0.26	0.013	−0.35	−0.14	0.20	−0.89–0.19		−0.56	0.014	−0.65	0.141	0.11	0.805
Presence of chronic disease	3.85	0.06	0.61	−10.94–18.65	0.003	8.70	0.13	0.24	−5.77–23.17		8.82	0.142	20.78	0.08	7.84	0.510
	**Males (*n* = 43)**															
												**Quantile regression**				
	**Simple linear regression model**						**Multiple linear regression model**					**25th**		**Median**		**75th**
**Independent variables**	**β**	**Standardized β**	***p*-Value**	**95% CI**	***R*^2^**	**β**	**Standardized β**	***p*-Value**	**95% CI**	***R*^2^**	**β**	***p*-Value**	**β**	***p*-Value**	**β**	***p*-Value**
BMI	0.52	0.12	0.46	−0.88–1.91	0.011	0.66	0.15	0.348	−0.74–2.06	0.087	0.50	0.486	0.97	0.218	−0.54	0.689
Age (years)	−0.483	−0.25	0.11	−1.09–0.12	0.060	−0.52	−0.26	0.095	−1.14–0.10		−0.31	0.320	−0.88	0.013	−0.38	0.528
Presence of chronic disease	2.96	0.06	0.72	−13.396–19.322	0.003	5.36	0.10	0.511	−10.95–21.69		2.25	0.786	8.36	0.361	9.10	0.567

**Table 3:** Odds ratio (OR) and 95% CIs for scoring above 75th percentile ORWELL 97 and among patients with obesity seeking treatment.

	**Females (*n* = 86)**					
	**Simple Logistic Regression Model**			**Multiple Logistic Model**		
Independent variables	OR	95% CI	*p*-Value	OR	95% CI	*p*-Value
BMI	1.22	1.08–1.38	<0.001	1.23	1.09–1.40	<0.05
Age (years)	1.01	0.97–1.05	0.629	1.01	0.97–1.05	0.574
Presence of chronic disease						
No	1.00			1.00		
Yes	1.22	0.469–3.23	0.692	1.37	0.44–4.22	0.589
	**Males (*n* = 43)**					
	**Simple logistic regression model**			**Multiple logistic model**		
Independent variables	OR	95% CI	*p*-Value	OR	95% CI	*p*-Value
BMI	0.94	0.81–1.09	0.417	0.98	0.85–1.13	0.754
Age (years)	0.92	0.84–1.01	0.078	0.92	0.84–1.01	0.075
Presence of chronic disease						
No	1.00			1.00		
Yes	2.50	0.51–12.18	0.257	2.85	0.50–16.23	0.239

## References

[1] Garratt A., Schmidt L., Mackintosh A., Fitzpatrick R. (2002). Quality of life measurement: Bibliographic study of patient assessed health outcome measures. BMJ.

[2] Testa M.A., Simonson D.C. (1996). Assessment of quality-of-life outcomes. N. Engl. J. Med..

[3] Kroes M., Osei-Assibey G., Baker-Searle R., Huang J. (2016). Impact of weight change on quality of life in adults with overweight/obesity in the United States: A systematic review. Curr. Med. Res. Opin..

[4] Collaborators GBDO (2017). Health Effects of Overweight and Obesity in 195 Countries over 25 Years. N. Engl. J. Med..

[5] Gregg E.W., Shaw J.E. (2017). Global health effects of overweight and obesity. N. Engl. J. Med..

[6] Stenholm S., Head J., Aalto V., Kivimaki M., Kawachi I., Zins M., Goldberg M., Platts L.G., Zaninotto P., Magnusson Hanson L.L. (2017). Body mass index as a predictor of healthy and disease-free life expectancy between ages 50 and 75: A multicohort study. Int. J. Obes..

[7] Ghosh R.K., Ghosh S.M., Ganguly G. (2010). Health-related quality of life and its growing importance in clinical practice. N. Z. Med. J..

[8] Kreidieh D., Itani L., El Kassas G., El Masri D., Calugi S., Dalle Grave R., El Ghoch M. (2017). Long-term Lifestyle-modification programs for overweight and obesity management in the Arab states: Systematic review and meta-analysis. Curr. Diabetes Rev..

[9] Al Sayah F., Ishaque S., Lau D., Johnson J.A. (2013). Health related quality of life measures in Arabic speaking populations: A systematic review on cross-cultural adaptation and measurement properties. Qual. Life Res..

[10] Ghroubi S., Elleuch H., Chikh T., Kaffel N., Abid M., Elleuch M.H. (2009). Physical training combined with dietary measures in the treatment of adult obesity. A comparison of two protocols. Ann. Phys. Rehabil. Med..

[11] Al Kadi A., Siddiqui Z.R., Malik A.M., Al Naami M. (2017). Comparison of the efficacy of standard bariatric surgical procedures on Saudi population using the bariatric analysis and reporting outcome system. Saudi Med. J..

[12] Itani L., Calugi S., Kreidieh D., El Kassas G., El Masri D., Tannir H., Dalle Grave R., Harfoush A., El Ghoch M. (2017). Validation of an Arabic version of the obesity-related wellbeing (ORWELL 97) questionnaire in adults with obesity. Curr. Diabetes Rev..

[13] Mannucci E., Ricca V., Barciulli E., Di Bernardo M., Travaglini R., Cabras P.L., Rotella C.M. (1999). Quality of life and overweight: The obesity related well-being (Orwell 97) questionnaire. Addict. Behav..

[14] Mannucci E., Petroni M.L., Villanova N., Rotella C.M., Apolone G., Marchesini G., QUOVADIS Study Group (2010). Clinical and psychological correlates of health-related quality of life in obese patients. Health Qual. Life Outcomes.

[15] Camolas J., Ferreira A., Mannucci E., Mascarenhas M., Carvalho M., Moreira P., do Carmo I., Santos O. (2016). Assessing quality of life in severe obesity: Development and psychometric properties of the ORWELL-R. Eat. Weight Disord..

[16] Mannucci E., Ricca V., Barciulli E., Di Bernardo M., Travaglini R., Cabras P.L., Rotella C.M. (2012). Obesity-related WELL-being questionnaire (ORWELL 97). Clinica Psicologica dell’Obesità.

[17] Choo J., Jeon S., Lee J. (2014). Gender differences in health-related quality of life associated with abdominal obesity in a Korean population. BMJ Open.

[18] Bentley T.G., Palta M., Paulsen A.J., Cherepanov D., Dunham N.C., Feeny D., Kaplan R.M., Fryback D.G. (2011). Race and gender associations between obesity and nine health-related quality-of-life measures. Qual. Life Res..

